# Student satisfaction and academic efficacy during online learning with the mediating effect of student engagement: A multi-country study

**DOI:** 10.1371/journal.pone.0285315

**Published:** 2023-10-04

**Authors:** Hamid Sharif Nia, João Marôco, Long She, Fatemeh Khoshnavay Fomani, Pardis Rahmatpour, Ivana Stepanovic Ilic, Maryam Mohammad Ibrahim, Fatima Muhammad Ibrahim, Sumit Narula, Giovanna Esposito, Ozkan Gorgulu, Navaz Naghavi, Saeed Pahlevan Sharif, Kelly-Ann Allen, Omolhoda Kaveh, Jonathan Reardon

**Affiliations:** 1 Educational Development Center, Mazandaran University of Medical Sciences, Sari, Iran; 2 Department of Nursing, Amol Faculty of Nursing and Midwifery, Mazandaran University of Medical Sciences, Sari, Iran; 3 William James Centre for Research ISPA–Instituto Universitário, Lisboa, Portugal; 4 FLU Pedagogy, Nord University, Bodø, Norway; 5 Sunway Business School, Sunway University, Sunway City, Selangor, Malaysia; 6 School of Nursing and Midwifery, Tehran University of Medical Sciences, Tehran, Iran; 7 Fellowship of e-learning in Medical Education, School of Nursing, Alborz University of Medical Sciences, Karaj, Iran; 8 Faculty of Philosophy, University of Belgrade, Beograd, Serbia; 9 Reproductive Health Department, School of Nursing and Midwifery, Tehran University of Medical Sciences, International Campus (TUMS-IC), Tehran, Iran; 10 Deputy Dean Research, Amity University Madhya Pradesh, Gwalior, India; 11 Department of Humanities, University of Naples Federico II, Napoli NA, Italy; 12 Department of Biostatistics and Medical Information, Faculty of Medicine, Kirsehir Ahi Evran University, Kirsehir, Turkey; 13 Faculty of Business & Law, Taylor’s University, Selangor, Malaysia; 14 School of Educational Psychology and Counselling, Faculty of Education, Monash University, Clayton, Australia; 15 Centre for Wellbeing Science, Melbourne Graduate School of Education, University of Melbourne, Parkvillle, Australia; 16 School of Nursing and Midwifery, Mazandaran University of Medical Sciences, Sari, Iran; 17 School of Education, Durham University, Durham, United Kingdom; Central China Normal University, CHINA

## Abstract

The COVID-19 pandemic caused unprecedented changes to educational institutions, forcing their closure and a subsequent shift to online education to cater to student learning requirements. However, successful online learning depends on several factors and may also vary between countries. As such, this cross-sectional study sought to investigate how engagement of university students, a major driver of online learning, was influenced by course content, online interaction, student acceptance, and satisfaction with online learning, as well as self-efficacy across nine countries (China, India, Iran, Italy, Malaysia, Portugal, Serbia, Turkey, and the United Arab Emirates) during the COVID-19 pandemic. Using a questionnaire-based approach, data collected from 6,489 university students showed that student engagement was strongly linked to perception of the quality of the course content and online interactions (p < .001). The current study also indicated that online interactions are a major determinant of academic efficacy but only if mediated by engagement within the online learning context. A negative correlation between student engagement and satisfaction with online learning was found, demonstrating the importance of students being engaged behaviorally, emotionally, and cognitively to feel satisfied with learning. Academic efficacy and student satisfaction were explained by course content, online interaction, and online learning acceptance, being mediated by student engagement. Student satisfaction and, to a lesser degree academic efficacy, were also associated with online learning acceptance. Overall, the structural equation model was a good fit for the data collected from all nine countries (CFI = .947, TLI = .943; RMSEA = .068; SRMR = .048), despite differences in the percentage variations explained by each factor (no invariance), likely due to differences in levels of technology use, learning management systems, and the preparedness of teachers to migrate to full online instruction. Despite limitations, the results of this study highlight the most important factors affecting online learning, providing insight into potential approaches for improving student experiences in online learning environments.

## Introduction

The lockdown, caused by the COVID-19 pandemic, forced many universities around the world to switch to online learning [1, 2]. Countries that had previously used online learning platforms had an advantage in how ready and able they were to transition. However, adjusting to online learning was a challenge for developing countries that lacked the required infrastructure and facilities. Students also faced personal challenges in needing to cope with, and adapt to, rapid change and uncertainty. While many college students in socioeconomically advantaged countries had prior experience, either with online learning, or with hybrid models that combined face-to-face learning with an online component [3], those in developing countries had little or no prior experience.

Success in online learning has been found to be dependent on several factors, such as the quality of university facilities, perceived support available, and teacher competence [4, 5]. One of the most important factors in online education is student engagement [6]. Students’ active engagement in online learning has been found to contribute to their academic achievement, learning performance, academic self-efficacy [7], and satisfaction with their overall university experience [8, 9].

Despite previously identified factors that point to successful online learning experiences in higher education, little research has examined cross-country differences. This study aims to investigate the relationships between online course content, online interactions, student acceptance, and satisfaction with online learning, as well as student self-efficacy and engagement in nine countries during the COVID-19 pandemic.

Lockdowns during the COVID-19 pandemic created challenges for traditional face-to-face education in most countries around the world. As a result, most schools and universities had to pivot course content and delivery to online platforms despite that the success of this approach was heavily dependent on the availability of country-specific facilities. For instance, China represented a country with significant pre-existing advances in online learning. Therefore, when over 220 million Chinese students were impacted by lockdown, the country responded with a well-structured response to organizing and managing online teaching [10–12]. On the other hand, India, with a high number of impacted students, considered to be a developing country, could not implement similar measures due to less information technology infrastructure and existing social and economic barriers. Serbia, with a smaller population, had over 85% of its teachers and students with existing internet access, IT facilities, and digital competencies, but reported a similar percentage to China and India of students needing to adopt online education during the pandemic [13]. In Iran, universities had reasonable existing infrastructure, experience, and acceptance towards online learning.

A concern with the closure of higher education institutions, was that for most countries, there was a need to respond rapidly which caused difficulties for those courses that required face-to-face training [14]. In Italy, for example, institutions reacted quickly. According to a survey carried out by the Conference of Italian University Rectors, 88% of the courses at Italian universities had been offered online since 24th March 2020 and half were offering 96% or more of their courses online after only a few days of lockdown (CRUI, 2020). During the COVID-19 pandemic, universities in Turkey began compulsory distance education in March 2020. Although 123 of Turkey’s 207 universities had distance-learning departments, with existing experience in distance education, they officially started their distance education processes on 23 March 2020 [15]. At Portugal, remote emergency online teaching was implemented up to two weeks after schools’ closure (March, 2020). However, lack of resources at students as well as of teachers’ homes have hindered the delivery of online lectures and assessments. Course contents were adapted for onsite classes, not online, students felt difficulties in interacting with colleagues and teachers alike in the content management systems–that are not truly e-learning of b-learning platforms–which correlated with poor e-leaning acceptance. The degree of cognitive and behavioral student engagement with the online learning seemed however, from informal teachers’ opinions, a key factor that was a potential mediator of students’ satisfaction with online learning as well as academic efficacy. This was also the case for Chinese student where a positive relationship between interaction and online learning satisfaction, interaction and academic self-efficacy, academic self-efficacy and student engagement, and the student engagement and online learning satisfaction were observed [16]. A recent review study by Zeng and Wang (2021) reinforced that due to individual differences, some students can do well in online courses while other students may not be able to do well in online courses. Course design that contributed to student online learning satisfaction suggested that instructors can proactively help student online learning by modifying online course components [17]. Familiarity with IT characteristics and complexity effects on e-learning satisfaction were mediated by online education perceived value [18]. There is growing evidence that appropriate facilities, including IT, course content, online interactions, and e-learning acceptance are an essential component of online learning and academic efficacy across countries. It remains however to be demonstrated if student engagement with online learning act as a mediator of course content, online interactions and e-learning acceptance effects on student satisfaction and academic efficacy, and if these effects are common to different countries across the world.

### Theoretical framework

#### Student engagement

Student engagement has been defined as the amount of physical and psychological effort that a student dedicates to learning [16, 19]. This suggests that student engagement is not only influenced by behavior but also by emotion and cognitive processes [20]. According to the Expectancy-value Theory proposed by Eccles et al. (1983), student ability, beliefs, task–specific expectancy and subjective task value (how the task meets the students different needs) can determine their achievement -related choice, behavior and persistence [21]. The Expectancy-value Theory can be used to consider the related factors of students’ behavioral engagement as well as the several factors that influence students’ values and beliefs [21, 22]. Students’ active engagement in learning is seen as central to their academic success, learning satisfaction, academic achievement, and completion rates [16, 23–25]. Studies have shown that more deeply engaged students are motivated to learn by intrinsic interest in the subject rather than by fear of failing assessments, and that they are more likely to understand what they have learned [26, 27]. Moreover, students who engage deeply with learning are better equipped for life-long learning [9]. In a separate development, Janosz [28] found that students must engage physically (i.e., behavioral engagement) and psychologically (i.e., emotional and cognitive engagement) in learning processes to acquire new skills and enhance academic capability. In the online learning environment, well-designed online learning activities have been found to harness student engagement through learner-centered instruction [29]. Other research has found self-efficacy to be critical to engagement [30]. Despite known factors that enhance engagement, some researchers have argued that it is difficult to develop student engagement in online learning; a lack of self-discipline or poor technological skills were found to be barriers [16, 31]. It is important to understand the determinants of student engagement in online contexts and its relationship with students’ academic self-efficacy given that both factors have an important role in online learning satisfaction.

#### Online learning satisfaction

Learning satisfaction is a multidimensional construct based on learners’ attitudes and feelings about their educational experience [16, 32]. In online learning, learners’ satisfaction was found to be a major factor affecting the continuity of online learning and learning effectiveness [33]. Previous studies on online learning have indicated that learner satisfaction is a major determinant of learning achievement and academic success [34]. Green et al. (2015) found that factors that affect learning could be divided into those related to teaching, the institution, and the learners themselves [35]. The teaching factors included communication and interaction with the instructor, lecturer empathy, management of the classroom, and instructor support of learning outside class. Institutional factors included peer support, course content support, and university facilities. Learner factors included learners’ personal characteristics, attitudes towards learning, knowing what supports self-learning, and intention to engage in academic activities [32]. The present study argues that course content quality and interaction (as teaching factors), online learning acceptance, and engagement in online classes (as learner’s factors) may contribute to students’ online learning satisfaction.

#### Academic self-efficacy

Self-efficacy refers to an individual’s confidence in their capabilities to organize and master a task to perform well [36]. In the same vein, academic self-efficacy represents an individual’s confidence that they can successfully execute academic tasks under designated circumstances [37]. As the main component of social cognitive theory, self-efficacy appears to be an important variable in the student learning process, and it significantly changes students motivation and learning [38]. Academic self-efficacy is closely linked with students’ favorable learning engagement, learning satisfaction, academic performance, and outcomes [16, 39]. Despite the important role of academic self-efficacy in educational research, the literature provides little insight into what can be done to drive students’ academic self-efficacy [38, 40]. Bandura (1986) had posited that self-efficacy can be developed through mastery experiences, vicarious experiences, social persuasions, and psychological states [41], with mastery experience being the most powerful source of developing a strong sense of efficacy as it provides students with authentic evidence of their competence [42]. Mastery experiences can also be linked to the students’ experiences of online learning, such as online learning quality, experience with new technology, and experiences with others during online learning Based on the theoretical evidence and the important role of academic self-efficacy in educational research, it is necessary to identify the factors that drive academic self-efficacy and provide recommendations for future research.

#### Students’ perception of course content

Course content generally refers to the topics, themes, concepts, or facts within a particular subject [43]. The quality of the course content can influence students’ knowledge and their ability to understand the subject matter, and hence, students who perceive that learning materials are meaningful to them will be more motivated to engage with their courses (often referred to as learner-to-content engagement) [44, 45]. The need for quality content takes on an even greater importance within the online learning environment where students are reportedly more prone to feelings of boredom or lack of engagement compared to face-to-face students [46]. Kumar et al. reported a significant positive relationship between online learning content and online learning quality, with the latter further impacting learner satisfaction [43]. Leire et al. (2016) suggested that successful online courses are those that have readily-available and diverse content that consider the diverse learning needs of students [47]. Similarly, Kauffman (2015) pointed out that students have a different perception of online courses compared with traditional face-to-face courses, and as such, they may more readily form negative opinions regarding poor online content which are not aligned with learning outcomes, leading to reduced motivation and persistence in learning [48]. While it is recognized that adapting discipline-specific content to online platforms can be particularly challenging, well-developed course content remains an important factor for sustaining learners’ interests in online learning.

#### Interactions between teachers and students

Positive interactions between teachers and students lead to student satisfaction and student learning outcomes, however, interactions between students and lecturers were significantly hindered during the pandemic through disrupted opportunities for face-to-face interactions and relationship building. Building relationships between students and lecturers can be beneficial for active engagement in classes [9]. In an online-context, discussions forums have been one way to facilitate positive interactions by helping students to feel comfortable and share their thoughts and ideas [46], but more traditional methods of developing student motivation and engagement, such as interactive classes, group activities and active learning, are difficult to emulate in online settings. Because interactions between lecturers and students has been noted as crucial for learning [9], understanding the role positive interactions play in student self-efficacy and engagement is important.

#### Online learning acceptance

Online learning can provide opportunities for students through access to learning materials and ease of communication and collaboration [49]. According to the technology acceptance model (TAM) by Davis (1989) [50], acceptance of using a particular technology is a crucial factor that drives usage behavior [51, 52]. In addition, the Unified Theory of Acceptance and Use of Technology (UTAUT) (Venkatesh et.al. 2003) [53], as described by Pham and Tran (2020), explained online learning acceptance as intention and behaviors of learner for using technology that is affected by university support, students’ computer competencies, infrastructure, course content and design, and learner collaboration [54]. Previous studies have suggested that acceptance of technology affects online learning outcomes, including online learning system success, its quality, and learning satisfaction [49, 55–58]. In the same vein, Cheng (2011) stated that acceptance of technology increases learners’ experience in total engagement with online learning. When individuals accept the online learning system, they are more focused on the learning process, the level of cognitive burden is reduced, they engage more with online learning activities, and gain more confidence in them [57]. Even though the important role of acceptance of technology has been addressed in previous studies, research on its role in higher education institutions is limited [59].

Following the exposed framework and the proposed theoretical relations between constructs and outcomes, and the Self-System Model and Expectancy-Value Theory, that course content, interaction with peers and teachers, and acceptance of online learning can impact student engagement which in turn can determine academic efficacy and lead to academic achievement [60, 61], we assessed the following hypothesis:

*H1*: *Course content is positively related to student online learning satisfaction*.
*H2*: *Course content is positively related to student self-efficacy*.
*H3*: *Interaction is positively related to student online learning satisfaction*.
*H4*: *Interaction is positively related to student self-efficacy*.
*H5*: *Online learning acceptance is positively related to student online learning satisfaction*.
*H6*: *Online learning acceptance is positively related to student self-efficacy*.
*H7*: *Student engagement is positively related to student online learning satisfaction*.
*H8*: *Student engagement is positively related to student self-efficacy*.
*H9*: *Student engagement is mediating the relationship between course content and student online learning satisfaction*.
*H10*: *Student engagement is mediating the relationship between course content and student self-efficacy*.
*H11*: *Student engagement is mediating the relationship between interaction and student online learning satisfaction*.
*H12*: *Student engagement is mediating the relationship between interaction and student self-efficacy*.
*H13*: *Student engagement is mediating the relationship between online learning acceptance and student online learning satisfaction*.
*H14*: *Student engagement is mediating the relationship between online learning* acceptance *and student self-efficacy*.

## Methods

### Design

This cross-sectional study included university students from China, India, Iran, Italy, Malaysia, Portugal, Serbia, Turkey, and the United Arab Emirates (UAE). Students were invited through online forums, email, and institutional sites, to answer a survey with a sociodemographic questionnaire and measures related to the model during the COVID-19 pandemic from April to August 2021.

### Participants

In this study, 6,489 university students from nine contrasting countries in Europe and Asia (see Table 1 for characterization of the participants) were included. A non-probabilistic snowball sampling method was used. Online data gathering was performed via Google forms and Microsoft Excel software was used to evaluate the accuracy and validity of the data. The questionnaire was prepared in seven languages: Chinese, Italian, Serbian, Portuguese, Persian, Turkish, and English. Questionnaire translations were performed by local experts in education, psychometrics, and their native language. Iranian university students responded to the Persian language questionnaire, and Turkish students chose one of the two Turkish and English language questionnaires. All other students answered the questionnaire in their native language. The URL links of questionnaires were sent to all university students without considering their field of study. The questionnaire links were first sent to students through social media groups (Telegram or WhatsApp), emails, and institutional websites, and then they were asked to share the questionnaire link with other classmates if they wished.

**Table 1:** Participants demographic information and Mean (SD) Z scores for each factor per country.

Variable	China, N = 1,504^*1*^	Iran N = 921^*1*^	Turkey N = 413^*1*^	Serbia N = 595^*1*^	India, N = 518^*1*^	Portugal, N = 1,172^*1*^	Malaysia N = 383^*1*^	Italy N = 465^*1*^	UAE N = 525^*1*^
**Gender**(N = 6,489)									
Female n(%)	446 (30%)	628 (68%)	265 (64%)	98 (16%)	180 (35%)	905 (77%)	164 (43%)	362 (78%)	390 (74%)
Male n(%)	1,058 (70%)	293 (32%)	148 (36%)	497 (84%)	333 (65%)	266 (23%)	218 (57%)	103 (22%)	135 (26%)
**Age** mean (range)	20.0 (19.0, 20.0)	21.0 (20.0, 23.0)	21.0 (20.0, 23.0)	21.0 (20.0, 23.0)	21.0 (19.0, 23.0)	21.0 (19.0, 25.0)	21.0 (20.0, 22.0)	22.0 (21.0, 24.0)	25.0 (21.8, 32.0)
**Degree**(N = 6,495)									
BSc n(%)	1,470 (98%)	888 (96%)	259 (63%)	532 (89%)	315 (61%)	912 (78%)	383 (100%)	282 (61%)	384 (73%)
MSc n(%)	18 (1.2%)	16 (1.7%)	27 (6.5%)	46 (7.7%)	160 (31%)	241 (21%)	0 (0%)	120 (26%)	112 (21%)
Doctorate n(%)	0 (0%)	5 (0.5%)	122 (30%)	0 (0%)	0 (0%)	0 (0%)	0 (0%)	60 (13%)	6 (1.1%)
PhD n(%)	16 (1.1%)	12 (1.3%)	5 (1.2%)	17 (2.9%)	43 (8.3%)	18 (1.5%)	0 (0%)	3 (0.6%)	23 (4.4%)
**Z score of factors**									
Course Content	0.343 (0.760)	-0.740 (0.775)	-0.292 (0.787)	0.065 (0.692)	0.162 (0.773)	0.084 (0.634)	0.199 (0.588)	-0.099 (0.492)	0.097 (0.778)
Interaction	0.358 (0.802)	-0.738 (0.850)	-0.292 (0.854)	0.109 (0.772)	0.183 (0.832)	0.043 (0.692)	0.168 (0.648)	-0.018 (0.551)	0.010 (0.823)
Student satisfaction	0.286 (0.770)	-0.614 (0.932)	-0.249 (0.941)	-0.065 (0.859)	0.098 (0.878)	-0.012 (0.860)	0.209 (0.731)	0.061 (0.671)	0.254 (0.838)
ELearning acceptance	0.055 (0.161)	-0.095 (0.197)	-0.044 (0.198)	-0.034 (0.199)	0.019 (0.184)	-0.012 (0.200)	0.021 (0.162)	0.023 (0.151)	0.053 (0.179)
Academic efficacy	0.301 (0.776)	-0.412 (0.775)	-0.133 (0.677)	0.194 (0.605)	0.176 (0.769)	-0.185 (0.723)	-0.041 (0.604)	0.047 (0.431)	0.015 (0.820)
Student engagement	0.207 (0.684)	-0.218 (0.612)	-0.005 (0.518)	0.114 (0.518)	0.226 (0.628)	-0.148 (0.546)	-0.136 (0.474)	0.006 (0.340)	-0.129 (0.694)

### Research instruments

The University Student Engagement Inventory (USEI) was developed by Maroco for Portuguese university students and was adapted for English, Italian, Serbian, Persian, and Chinese students [20, 24, 62]. It consists of 15 items and 3 subscales, including behavioral, emotional, and cognitive engagement. The USEI is scored on a 5-point Likert-type scale from 1 (never) to 5 (always), and a reversed scoring method was used for one negative question (item 6: “I don’t feel very accomplished at this school”). The range of scores for each of the subscales was between 5 and 25and higher scores indicated higher student engagement [20]. The sample items include “I usually do my homework on time” (Behavioral engagement), “I am interested in the school work” (Emotional engagement), and “I try to integrate the acquired knowledge in solving new problems” (Cognitive engagement).

Self-efficacy is a subscale of academic burnout questionnaires developed by Hu & Schaufeli [63]. This subscale has six items with 5-point Likert scores ranging from “strongly disagree” to “strongly agree”. Sample items of self-efficacy include “I can effectively solve the problems that arise in my studies”.

Interaction and Course content are the subscales of student perceptions of an online course (SPOC) scale. The SPOC scale was developed by Chung & Chen [64]. The *interaction subscale* has six items such as “the instructor encourages student participation and questions” and “the course content provides mutual interaction to facilitate student learning”. A 5-point Likert rating scale scored from 1 (strongly disagree) to 5 (strongly agree) was used. *Course content* questions were about students’ perception of course content quality in online learning. Respondents were asked to respond on a five-point Likert scale ranging from 1 (strongly disagree) to 5 (strongly agree) to each statement (e.g., “The course content appears to be current for the subject matter presented”).

Satisfaction to online learning was measured by instruments developed by Lee [65], and online learning acceptance or usefulness questions was part of Pham & Tran (2020) study questionnaire [54]. These items with 5-point Likert scores ranging from “strongly disagree” to “strongly agree”. Sample item of satisfaction and acceptance were “This online course met my needs as a learner” and “online learning is a failure and a bad idea” respectively.

### Data analysis

Descriptive statistics per item and country were obtained with the R packages gtsummay [66] and skimr [67]. Skewness (sk) and kurtosis (ku) measures were used to determine if the data deviated from normality. Absolute values of sk and ku below 3 and 7 were deemed adequate for further confirmatory analysis using standard and robust estimation methods [68]. Evidence of validity and reliability were gathered with confirmatory factor analysis on the polychoric correlation matrix using the DWLS estimator present in the R package lavaan [69]. The hypothetic causal model fit was tested on the polychoric correlation matrix of all the items in all countries using the DWLS estimator present in lavaan. The goodness of fit of both confirmatory factor models and structural model was evaluated using the Confirmatory Fit Index (CFI), the Tucker-Lewis index (TLI), the Root Mean Square Error of Approximation (RMSEA), and the Standardized Root Mean Residual (SRMR). The models’ fit to the data was deemed good for CFI and TLI above 0.9 and for RMSEA and SRMR below 0.06 and 0.08 respectively [70, 71].

Evidence of reliability was assessed with the McDonald’s ω for first order factors and ω_L1_ for second order factors using the R package SemTools [72]. Omegas greater than 0.7 was indicative of good reliability [73]. Test of the SEM model configural and metric invariance for countries was tested by comparing a set of nested models with free loadings (configural invariance), constrained loadings to be equal between countries (metric invariance), and constrained factor loadings and intercepts (scalar invariance). Invariance was accepted for non-significant Δχ2 between consecutive constrained models (p>0.05) and for absolute ΔCFI smaller than 0.01 [74]. To avoid overweight of countries with large sample sizes in the invariance analysis, a random sample of 500 participants was drawn for countries with sample sizes larger than 500, and the combined samples were used for further analysis of invariance.

The protocol for this study was approved by Mazandaran University of Medical Sciences (IR.MAZUMS.REC.1399.7523). The study aims, number of items, time to complete the survey, the researcher’s affiliation and email for queries, and the ethical code of study were included on the first page of the online questionnaire. Participants were informed that their participation was voluntary and that their responses would be published anonymously as group data. The online questionnaire items are not viewed by participants until they agree to participate by clicking on the “next” button. Clicking this button also signaled completion of the informed consent form.

## Results

### Descriptive analysis

Summary measures for participants are presented in Table 1. Overall participants’ median age was 21 (IQR = 3) years with younger students in China (Md = 20) and older students in the United Arab Emirates (UAE) (Md = 25). Fifty-three percent were females and 47% were males. Eighty-four percent of students were enrolled in a BSc degree, 11% in an MSc degree, and 5% in a doctorate/PhD degree.

In addition, Z scores were estimated from the overall CFA model using the WLSMV estimator. Based on these results, the lowest scores in each factor were for Iranian students. Chinese students received the highest score on each factor except student engagement. Student engagement has the highest score among Indian students (Mean = 0.226 and SD = 0.628). See Table 1.

### Measurements instruments evidence of validity and reliability

Confirmatory factor analysis of the instruments used in the structural model was used to gather evidence of the factors’ validity and reliability. Table 2 summarizes the range of standardized factor loadings (l), measures of goodness of fit, and reliability (ω). All first order and second-order factors displayed good evidence of factor validity and reliability.

**Table 2:** Standardized factor loadings (λ) range, goodness of fit (CFI, TLI, RMSEA, SRMR), and reliability (ω, ω_L1_) for the first order and second-order factors of the measuring instruments.

Instrument	Standardized factor loadings range	CFI	TLI	RMSEA	SRMR	ω	ω_L1_
**Perceptions of Online Course Contents (SPOC)**	.920-.980	0.998	0.997	0.064	0.029		0.934
Course Content	.713-.895					0.941	
Interaction	.843-.904					0.917	
**Student Satisfaction**	.939-.939	0.999	0.999	0.000	0.000	0.907	
**Online learning Acceptance or Usefulness**	.282-.941	0.999	0.999	0.000	0.000	0.748	
**Academic Efficacy**	.738-.840	0.996	0.994	0.071	0.035	0.868	
**University Support and Acceptance**	.662-.840	0.996	0.994	0.071	0.035	0.868	
**University Student Engagement Inventory**	.800-.880	0.987	0.984	0.089	0.060		0.823
Behavioral Engagement	.727-.767					0.823	
Emotion Engagement	.733-.894					0.851	
Cognitive Engagement	.736-.904					0.870	

### Structural equation model of academic efficacy and student satisfaction

**Fig 1:**
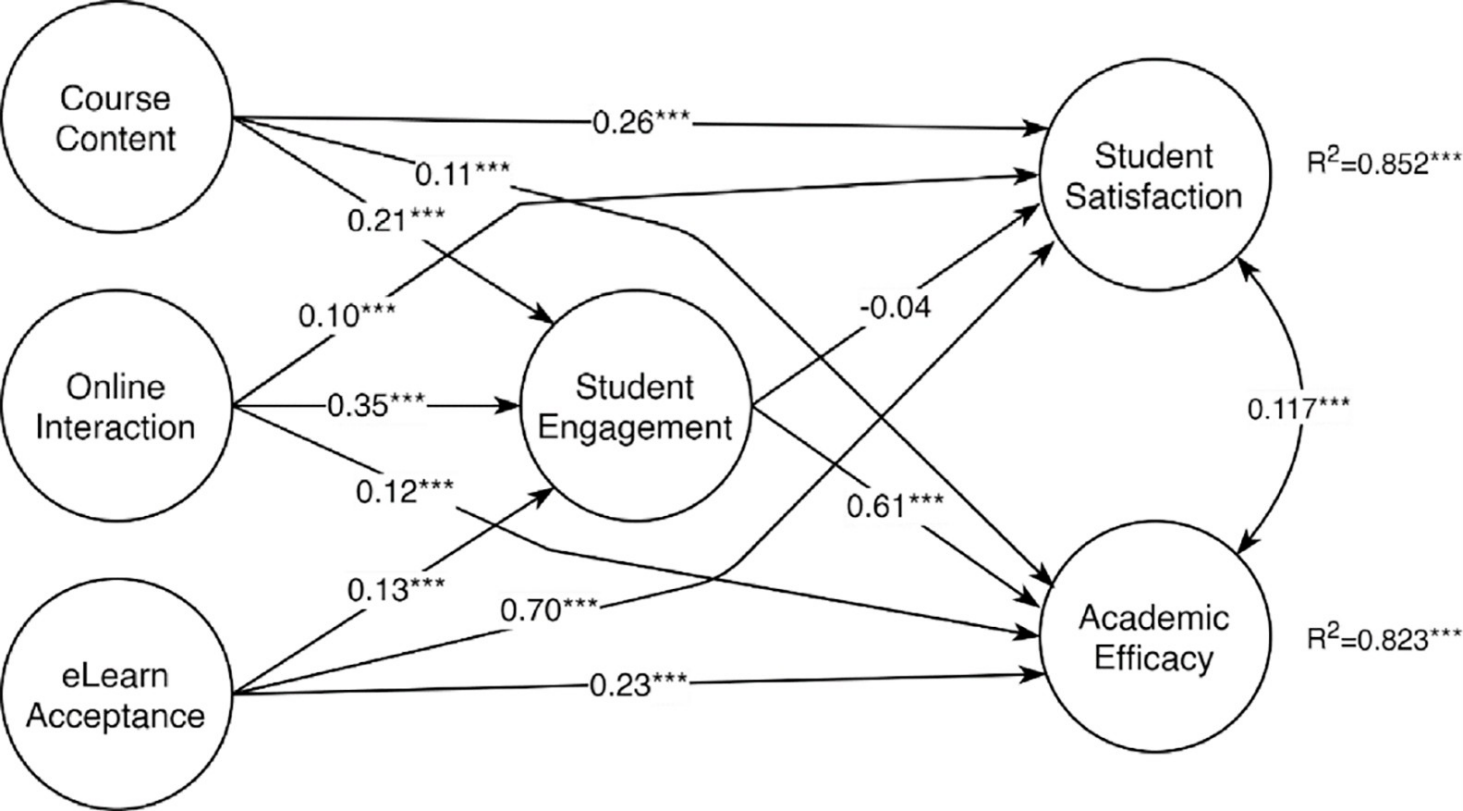
Academic efficacy and student satisfaction on course contents, online interaction, and online learning acceptance mediated by student engagement on the nine countries’ sample. Values are the standardized structural coefficients. ***—p < .001; R^2^ is the coefficient of determination.

Fig 1 summarizes the standardized structural coefficients for the regression of academic efficacy and student satisfaction on course contents, online interaction, and online learning acceptance mediated by student engagement in the nine-country sample. The model had a good fit to the data of the nine countries participating in this study (CFI = .947, TLI = .943; RMSEA = .068; SRMR = .048). The larger effect of students’ perception of the quality of course contents was on student satisfaction (β = 0.263; z = 14.218, p < .001) followed by student engagement (β = 0.209; p < .001). The major effect of students’ online interaction was on student engagement (β = 0.209, p < .001). Finally, online learning acceptance had strong implications for student satisfaction (β = 0.699; p < .001) and on academic efficacy, although to a lesser extent (β = 0.231; p<0.001). Student engagement was a strong mediator for course content (indirect effect = 0.228; p<0.001) and online interaction (indirect effect = 0.213, p<0.001) but less for online learning acceptance (indirect effect = 0.030, p < .001). Student engagement has no relevant effect on student satisfaction, but its mediating effects on course contents and online interaction were stronger than the direct effects of these variables on academic efficacy.

### Invariance of the structural equation model of academic efficacy and student satisfaction among nine countries

The analysis of invariance revealed that the configural invariance (same model structure) holds for all the countries (CFI = 0.904, RMSEA = 0.061) but no metric invariance (same structure and factor loadings) was observed between the countries (Δχ^2^(231) = 2750.174, p<0.001; ΔCFI = -0.027). Table 3 presents the standardized factor loadings for the nine countries in the sample. Explained variation for student satisfaction ranged from 64.5% in the UAE up to 95.8% in China. For academic efficacy, models explained from 65.2% in the UAE up to 98.8% in India, of the countries’ total variation.

**Table 3:** Standardized structural coefficients for the countries and R2 for criterion variables.

Country	Predictor		β	^ ^		Criterion	R^2^
China	StdEng	–	-0.018	^ns^	→	StdSat	0.958
	CourseContent	–	0.225		→		
	OnlineInter	–	-0.080	^ns^	→		
	eLearnAccept	–	0.889	^ns^	→		
	StdEng	–	0.738	***	→	AcadEff	0.888
	CourseContent	–	0.158	**	→		
	OnlineInter	–	0.064	^ns^	→		
	eLearnAccept	–	0.037	^ns^	→		
	CourseContent	–	0.350	***	→	StdEng	0.624
	OnlineInter	–	0.510	***	→		
	eLearnAccept	–	-0.015	^ns^	→		
Iran	StdEng	–	0.066	^ns^	→	StdSat	0.884
	CourseContent	–	0.177		→		
	OnlineInter	–	0.150	^ns^	→		
	eLearnAccept	–	0.731	***	→		
	StdEng	–	1.458	^ns^	→	AcadEff	0.974
	CourseContent	–	-0.014	^ns^	→		
	OnlineInter	–	-0.050	^ns^	→		
	eLearnAccept	–	-0.524	^ns^	→		
	CourseContent	–	0.103	^ns^	→	StdEng	0.949
	OnlineInter	–	0.123	^ns^	→		
	eLearnAccept	–	0.875	***	→		
Turkey	StdEng	–	-0.038	^ns^	→	StdSat	0.859
	CourseContent	–	0.308		→		
	OnlineInter	–	0.158	^ns^	→		
	eLearnAccept	–	-0.550	***	→		
	StdEng	–	0.491	***	→	AcadEff	0.744
	CourseContent	–	-0.058	^ns^	→		
	OnlineInter	–	0.349		→		
	eLearnAccept	–	-0.287	***	→		
	CourseContent	–	0.499		→	StdEng	0.243
	OnlineInter	–	0.002	^ns^	→		
	eLearnAccept	–	0.053	^ns^	→		
Serbia	StdEng	–	-0.049	^ns^	→	StdSat	0.843
	CourseContent	–	0.162	^ns^	→		
	OnlineInter	–	0.216	^ns^	→		
	eLearnAccept	–	0.648	***	→		
	StdEng	–	0.424	***	→	AcadEff	0.822
	CourseContent	–	-0.052	^ns^	→		
	OnlineInter	–	0.436		→		
	eLearnAccept	–	0.216	**	→		
	CourseContent	–	0.414	^ns^	→	StdEng	0.487
	OnlineInter	–	0.155	^ns^	→		
	eLearnAccept	–	0.196	^ ^	→		
India	StdEng	–	0.016	^ns^	→	StdSat	0.859
	CourseContent	–	0.096	^ns^	→		
	OnlineInter	–	0.098	^ns^	→		
	eLearnAccept	–	0.891	***	→		
	StdEng	–	0.983	^ns^	→	AcadEff	0.988
	CourseContent	–	0.143	^ns^	→		
	OnlineInter	–	-0.139	^ns^	→		
	eLearnAccept	–	0.076	^ns^	→		
	CourseContent	–	0.302	^ns^	→	StdEng	0.278
	OnlineInter	–	0.229	^ns^	→		
	eLearnAccept	–	-0.040	^ns^	→		
Portugal	StdEng	–	-0.036	^ns^	→	StdSat	0.777
	CourseContent	–	0.337	***	→		
	OnlineInter	–	0.125		→		
	eLearnAccept	–	-0.569	***	→		
	StdEng	–	0.347	***	→	AcadEff	0.775
	CourseContent	–	0.225	**	→		
	OnlineInter	–	0.129	^ns^	→		
	eLearnAccept	–	-0.497	***	→		
	CourseContent	–	0.429	**	→	StdEng	0.164
	OnlineInter	–	0.049	^ns^	→		
	eLearnAccept	–	0.170	**	→		
Malaysia	StdEng	–	-0.053	^ns^	→	StdSat	0.728
	CourseContent	–	0.538	***	→		
	OnlineInter	–	-0.044	^ns^	→		
	eLearnAccept	–	0.477	***	→		
	StdEng	–	0.283	***	→	AcadEff	0.745
	CourseContent	–	0.040	^ns^	→		
	OnlineInter	–	0.431		→		
	eLearnAccept	–	0.301	***	→		
	CourseContent	–	0.102	^ns^	→	StdEng	0.32
	OnlineInter	–	0.506		→		
	eLearnAccept	–	-0.067	^ns^	→		
Italy	StdEng	–	0.040	^ns^	→	StdSat	0.763
	CourseContent	–	0.171		→		
	OnlineInter	–	0.076	^ns^	→		
	eLearnAccept	–	0.690	***	→		
	StdEng	–	0.643	**	→	AcadEff	0.813
	CourseContent	–	0.336	***	→		
	OnlineInter	–	-0.077	^ns^	→		
	eLearnAccept	–	-0.056	^ns^	→		
	CourseContent	–	0.331	**	→	StdEng	0.288
	OnlineInter	–	0.265		→		
	eLearnAccept	–	-0.095	^ns^	→		
UAE	StdEng	–	0.039	^ns^	→	StdSat	0.645
	CourseContent	–	0.171	*	→		
	OnlineInter	–	0.076	^ns^	→		
	eLearnAccept	–	0.69	***	→		
	StdEng	–	0.643	**	→	AcadEff	0.652
	CourseContent	–	0.336	***	→		
	OnlineInter	–	-0.077	^ns^	→		
	eLearnAccept	–	-0.056	^ns^	→		
	CourseContent	–	0.331	**	→	StdEng	0.288
	OnlineInter	–	0.265	*	→		
	eLearnAccept	–	-0.095	^ns^	→		

* StdEng–Student Engagement; StdSat–Student Satisfaction; AcadEff–Academic Efficacy

## Discussion

The current study sought to identify the critical factors influencing student academic efficacy and satisfaction with participation in online courses during the COVID-19 pandemic. We also examined the mediating role of student engagement in the relationship between online course content, online interaction, student acceptance, student satisfaction, and student academic efficacy. The COVID-19 pandemic affected countries all over the world in a different manner but with a common response—promoted by the World Health Organization—to close schools, workplaces, and international borders to contain the SARS-CoV-2 spread. While the effects of schools’ lockdown on student learning remains to be seen, it was clear from the start that new online or hybrid learning environments brought new challenges for teaching and learning at different levels of the educational system. Not surprisingly, during the COVID-19 pandemic, studies revealed student dissatisfaction with online learning [75] and negative attitudes towards it [76]. The degree to which students are satisfied with online learning is determined by a variety of factors in different personal, course, and institutional aspects. Accordingly, student capability, skills, and health status, in addition to the degree of support they receive from teachers, can determine satisfaction with online learning [75, 77]. These factors can also affect student engagement [78]. Student engagement with online learning, perception of the quality of course content, and online interactions with teachers and peers were identified as key factors of learning and academic efficacy in studies of online learning before the COVID-19 pandemic [52, 79, 80]. In this study, we report on how a large sample of university students from nine countries (in Europe, Asia, and the Middle East) were affected by the pandemic. In particular, we describe how student engagement with online learning mediated perceptions of student satisfaction and academic efficacy as well as the quality of both course content and online interactions with peers and colleagues during the online emergency teaching adopted by participating countries.

The overall model fit to the combined data of the nine participating countries suggested a common trend for the mediating effect of student engagement with online teaching on academic efficacy but not on student satisfaction. Student satisfaction with online teaching was primarily explained by the acceptance of online learning and by the quality of course content. Student engagement with online learning was influenced by the satisfaction and frequency of online interaction. These results add to previous observations on online instruction contexts related to the COVID-19 lockdowns, particularly with regard to student satisfaction, academic efficacy, and learning [4]. Existing studies mostly highlight the quality of online learning derived from service quality, teachers’ roles, and the overall system quality as major determinants of student’ satisfaction and perceived competence in online environments, while students’ digital competencies and online interactions were considered to be slightly less important factors.

In this study, we show that online interactions are a major determinant of academic efficacy, but only if mediated by engagement within the online learning context. In line with the current findings, other studies have indicated that the more students interact with their teachers or peers, the higher their scores on academic efficacy scales [81, 82]. According to the Self-System Model and Expectancy-Value Theory, student engagement can determine academic efficacy, leading to later academic achievement [60, 61]. Both models propose that student engagement indicators can modulate the relationship between self-efficacy and achievement. Considering the engagement concept analysis, behavioral and emotional dimensions of engagement are considered key factors of a functioning school. While the behavioral dimension refers to student observable actions in the class, emotional engagement presents the students’ affective reactions to the teaching environment and learning activities [20]. Despite the links between these two dimensions, engaging emotionally or behaviorally in some learning activities does not necessarily lead to academic achievement [83]. Considering the Self-System Model and Expectancy-Value Theory, if student engagement plays a positive role, academic achievement will be the outcome of self-efficacy [60, 61].

It has been suggested that there is a negative correlation between student engagement and satisfaction with online learning. This finding shows that despite some studies demonstrating a mediating role of students’ engagement in the relationship between students’ interaction and satisfaction [84], their engagement in online learning will not necessarily lead to satisfaction with this type of education. Namely, student engagement is a multidimensional construct that includes behavioral, emotional, and cognitive domains [20, 85]; it cannot be achieved unless all three domains of engagement are met physically (behavioral) and psychologically (emotional and cognitive) [28]. If students fail to engage in one or all domains, it is expected that they will experience low levels of learning satisfaction [23, 86].

We also found that course content and online learning acceptance are positively correlated with student satisfaction and academic efficacy, while student engagement plays a mediator role. These findings are supported by the existing literature [87–89]. Regarding course content, studies have demonstrated some aspects of the course content, such as consistency and density [90] and flexibility and quality [91], influence student satisfaction. According to the Technology Acceptance Model (TAM) by Davis (1989) [50], course design and content can be considered as factors that lead to online learning success. Some factors such as perceived usefulness and ease of use of online technology have been identified as the predecessor to online learning success [92].

A plethora of papers have demonstrated a positive relationship between student engagement and academic efficacy [16, 82]. Our results fall in line with past work, providing evidence that student engagement is the driving force of academic efficacy in an online learning environment across nine different countries. That is, student engagement potentiates the effects of course content quality and online interactions on the students’ perceived academic efficacy. Engaged students display higher perceived academic efficacy than non-engaged students [16, 20, 93]. These results suggest that online teaching in schools must promote student engagement as a strategy to facilitate better quality online learning.

Despite the significant and overall large effects on predicting academic efficacy and student satisfaction with online instruction (R^2^ larger than 80%), the model is not invariant in all countries. This lack of invariance can be attributed to different levels of technology use and learning management systems before the pandemic (e.g., Moodle and Blackboard) and the preparedness of teachers to migrate to full online instruction. In countries like Portugal or Italy, all universities had implemented LMS systems several years before the COVID-19 pandemic. Thus, a small learning effort was required to transfer to an online delivery of content and instruction. On the contrary, difficulties with home access to the internet (both speed and reliability) caused frustration among students and teachers alike, reducing engagement with the online instruction (see Italy and Portugal in Table 3). In other countries, such as Iran, the educational system was not prepared enough to conduct online courses as it takes time to transform traditional educational settings to learning management systems such as NAVID, VESTA, and Moodle. Studies indicate that there are several challenges involved, such as teachers’ long, monotonous monologues, lack of student participation, logistical problems, and carelessly chosen, repetitive tasks [94], as well as a lack of access to all infrastructures and equipment [14], that can affect Iranian student engagement. Online learning acceptance by Iranian students was the only predictor of student engagement with online classes (see Iran in Table 3).

While the overall structural model of student engagement as a mediator of student perceived academic self-efficacy fits nicely to our data, there were strong differences between countries on the observed effects of predictors on engagement and academic efficacy. These can be explained by face value attributed by students and families to higher education, cultural, educational, and social norms, access to online learning technology and resources that differed greatly in countries like Iran, the UAE, China, India, Turkey, Malaysia, Italy, Serbia or Portugal.

### Implications

The findings of the current study provide insight for education policymakers and teachers to promote the current necessity of online learning systems. Existing knowledge regarding online learning emphasizes the importance of factors that improve the student’s experience of online learning. Although countries’ experiences of online learning during the COVID-19 pandemic differed, the results of the present study still emphasize the importance of empowering students to improve their e-competencies to be active learners. Providing an educational atmosphere that promotes student engagement and selecting suitable course content, as well as improving online interactions, will lead to students’ academic efficacy. Student engagement can play a critical role in exploring the relationship between course content, online interaction, online learning acceptance, and academic efficacy. These conclusions have both theoretical and practical implications for educational researchers–who need to account student engagement in predictive models on academic efficacy and student satisfaction; as well as to higher education officials who need to implement policies promoting the engagement of students with their course work.

### Limitations

This study is not without limitations. Self-report measures may be subject to exaggeration due to social desirability bias. Also, the use of a cross-sectional research design cannot guarantee valid causal inferences. Causal hypotheses are supported by correlations, but the reverse may not be true. Furthermore, non-probabilistic snowball sampling does not guarantee the representativeness of the study populations. However, the large sample sizes per country (and overall) may appropriately represent the natural variation of the population and be adequate for hypothetical causal models. Future research may adopt longitudinal or experimental designs to provide more supporting evidence about the observed relationships and their underlying mechanisms. Additionally, it has been indicated that institutional factors such as openness to change, preparing educational technology, the institute culture and climate may have a direct (or indirect) impact on online learning [95]. While the current study focused on the proposed model (Fig 1), we suggest that further studies are conducted to investigate how institutional factors relate to both teaching and learner’s factors.

## Conclusions

This cross-sectional study investigated the main factors that influence students’ academic efficacy and satisfaction when they participated in online courses during the COVID-19 pandemic. Interestingly, data obtained from nine different countries displayed a similar trend, i.e., with student engagement being a strong mediator of the relationship between online teaching, especially online interactions and academic efficacy. Positive correlations between course content, online -learning acceptance, student satisfaction, and academic efficacy were also mediated by engagement levels of the students. However, the percentage variation explained by each factor was different across countries, and this could be attributed to existing differences in educational systems and social and cultural norms. The forced transition to online learning due to the pandemic undoubtedly highlighted a number of benefits along with key challenges associated with virtual education. Even as countries strive to return to normality, it is quite likely that online education will remain an important component of the learning system, and hence, it can be expected that the results of this study will be of relevance to educators as well as policymakers in view of enhancing students’ experiences of online learning.

## Supporting information

S1 Data (SAV)
